# Handoffs, safety culture, and practices: evidence from the hospital survey on patient safety culture

**DOI:** 10.1186/s12913-016-1502-7

**Published:** 2016-07-12

**Authors:** Soo-Hoon Lee, Phillip H. Phan, Todd Dorman, Sallie J. Weaver, Peter J. Pronovost

**Affiliations:** Strome College of Business, Old Dominion University, Norfolk, VA USA; Carey Business School, Johns Hopkins University, 100 International Drive, Baltimore, MD 21202 USA; School of Medicine, Johns Hopkins University, Baltimore, MD USA

**Keywords:** Handoffs, Staff attitudes, Patient safety culture, Communication, Personal responsibility, Accountability

## Abstract

**Background:**

The context of the study is the Agency for Healthcare Research and Quality’s Hospital Survey on Patient Safety Culture (HSOPSC). The purpose of the study is to analyze how different elements of patient safety culture are associated with clinical handoffs and perceptions of patient safety.

**Methods:**

The study was performed with hierarchical multiple linear regression on data from the 2010 Survey. We examine the statistical relationships between perceptions of handoffs and transitions practices, patient safety culture, and patient safety. We statistically controlled for the systematic effects of hospital size, type, ownership, and staffing levels on perceptions of patient safety.

**Results:**

The main findings were that the effective handoff of information, responsibility, and accountability were necessary to positive perceptions of patient safety. Feedback and communication about errors were positively related to the transfer of patient information; teamwork within units and the frequency of events reported were positively related to the transfer of personal responsibility during shift changes; and teamwork across units was positively related to the unit transfers of accountability for patients.

**Conclusions:**

In summary, staff views on the behavioral dimensions of handoffs influenced their perceptions of the hospital’s level of patient safety. Given the known psychological links between perception, attitude, and behavior, a potential implication is that better patient safety can be achieved by a tight focus on improving handoffs through training and monitoring.

**Electronic supplementary material:**

The online version of this article (doi:10.1186/s12913-016-1502-7) contains supplementary material, which is available to authorized users.

## Background

Clinical handoffs, also known as sign-outs, shift reports, or handovers, occur in many places along the healthcare value chain. It involves the ‘transfer of professional responsibility and accountability for some or all aspects of care for a patient, or groups of patients, to another person or professional group on a temporary or permanent basis’ [1]. For example, nursing handovers occur very frequently, not only between shifts and among part-time nurses, but also because nurses serve as the communication partner and informal coordinator for all healthcare professionals to ensure the continuity of care in a 24-hour seven-days-a-week environment [2]. The transfer of professional responsibility became salient for residents due to increased work-hour restrictions in U.S. residency programs, which shortened the continuity of care and increased the number of shift changes [3]. Concern for the transfer of unit accountability heightened with the fragmentation in the healthcare to the proliferation of sub-specialties; creating more transitions and handoffs with the increase in number of providers for a single patient [4]. Consequently, handoffs are a target for quality improvements because they represent high-risk events. The Joint Commission’s 2006 evaluation of accredited healthcare organizations attributed at least 35 % of sentinel events to handoff errors [5]. Recent estimates implicate handoff errors in nearly 80 % of serious events between 2004 and 2014 [6].

Patient safety culture, which consists of shared norms, values, behavioral patterns, rituals, and traditions [7] that guide the discretionary behaviors of healthcare professionals matter in handoffs. According to the theory of planned behavior [8], staff observations of their institution’s practices and coworkers’ behavioral patterns in handoffs will influence their perceptions of overall level of patient safety, and their behavioral responses to such issues. Therefore, employees who perceive that their do institutions not emphasize patient safety may not pay attention to such concerns [9]. To make improvements in handoffs, healthcare policymakers must first understand how employees perceive their organizations’ patient safety culture [10].

The extant literature on handoffs largely focuses on the relationship between inadequate communications and perceptions of avoidable harm [11–13]. Poor handoff communication creates an opportunity for adverse events because incomplete, inaccurate, and omitted data create ambiguities between the sending and receiving providers [14]. Yet, the literature has found little empirical evidence to suggest that effective information transfers are associated with positive perceptions of patient safety [15]. We surmise that this is because a handoff is multidimensional, involving the transfer of information, responsibility *and* accountability, implying that previous studies may have over-simplified handoff challenges [16].

This study contributes to the literature by empirically investigating what past research has largely ignored: the transfers of professional responsibility and unit accountability for patient safety between providers during handoffs [17]. In the transfer of responsibility, even with effective information exchange, whether the receiving provider feels the same sense of responsibility for the patient as the sending provider cannot be taken for granted. In the case of physicians, this sense of responsibility is defined by Horwitz and colleagues [18] as a sense among on-call physicians that they were not “just covering” for the admitting physician but rather are integral to the patient’s care. A systematic review on the transfer of information during nurses’ transitions of care found that senders exhibited few supportive behaviors during the shift change, resulting in a low degree of engagement by receivers as they demonstrated indifference and non-attentive behaviors [19]. Hence, we believe that during shift changes, the active role and the responsibility of healthcare providers in shaping an effective information exchange protocol go beyond the mere transmission of structured data [13, 16]. Without the effective transfer and acceptance of responsibility, there is no assurance that the handoff process has created an appropriate mental model of the patient’s plan of care for the receiving provider.

Our search of the literature did not yield any research on how the transfer of unit accountability influences staff perceptions of patient safety. Between-unit transitions of care can create uncertainty over who is ultimately accountable for a patient’s wellbeing. The cross-disciplinary and multi-specialty transition of care create coordination difficulties, as handoffs can be irregular and unpredictable [20, 21]. In addition, complications related to inter-professional differences in expectations, terminologies, and work practices make it challenging to build a shared mental model, necessary for effective transitions between providers [14]. Because conflicting expectations and perspectives between units increase barriers to effective handoffs, we expect that when healthcare professionals perceive a supportive environment for cooperation and joint accountability between units, they are more likely to have positive perceptions of patient safety.

We further expect handoffs of information, responsibility, and accountability to influence each other, so that improvement in one type will positively affect the other types, and degradation in one will erode the others. Specifically, handing off comprehensive and accurate patient information to a receiver is necessary for effectively handing off responsibility and accountability [22]. In a handoff, the failure of a sending unit to communicate the rationale for a decision, anticipate problems, and expectations creates uncertainties and ambiguities for the receiving unit [23]. Important information can be ignored or misinterpreted by the receiving unit when there is unclear handoff of responsibility and accountability resulting from ambiguous work procedures and a lack of supportive infrastructure [12].

We explore the factors in an organization’s patient safety culture that might be associated with effective handoffs. Specifically, we posit that an organization’s communication, teamwork, reporting, and management cultures will have differential influences on effective handoffs of information, responsibility, and accountability. The literature on information transfer has primarily dealt with the *mechanics* of communication (i.e., ways in which information is transmitted and received). We submit that this perspective is not complete without considering Marx’s theory of *just culture* [24]. Research has shown that when providers feel supported and psychologically safe because their organizations are perceived to be fair, they are more likely to communicate completely by voicing safety concerns [25, 26]. For example, in studies on TeamSTEPPS, a teaming protocol often used in surgical teams, any member (surgeon, nurse, technician, and anesthesiologist) can speak up or call-out observations of potential error because they view each other as having equal responsibility and authority for patient safety [27]. Feedback loops between the sender and receiver are necessary for this process to work. They allow both parties to properly manage expectations and adjust their behaviors. Hence, a strong communications culture, typified by the openness to and willingness of clinicians to speak up, ask questions, and provide feedback, would enhance effective handoff of information.

In the case of shift changes, a culture of professionalism can mitigate errors and procedural violations that arise primarily from aberrant mental processes such as forgetfulness, inattention, low motivation, carelessness, or negligence [28, 29]. Medical professionalism includes a commitment to collaborating with others while engaging in self-regulation to make the best clinical decisions [30]. Professionalism in nursing focuses on value-based cognitive and attitudinal attributes that are harnessed to deliver patient centered care [31]. Nurses often utilize handoffs as an avenue for socialization, education, and emotional support to facilitate integration and staff cohesion [19]. A teamwork culture facilitates handoff of responsibility between the sending and receiving providers by seeking assistance or voicing concerns and clarifying issues through bidirectional conversations. This process creates a *shared* mental model of the patient’s clinical conditional and plan of care [32]. Professionalism also implies proactive surveillance, detection, and the voluntary reporting of adverse events [33]. Errors recurrences are reduced if medical incidences and pitfalls are proactively reported to the incoming provider during shift changes [34]. Therefore, a strong teamwork culture and a culture of reporting adverse events enhance effective handoff of personal responsibility in shift changes.

Patient transfers between units span three domains: provider, service, and location, which are accompanied by differences in social norms, terminologies, and work practices [14, 18]. Such transitions multiply the difficulties providers encounter when building a shared mental model of the patient’s clinical problems and needs. Add to these are systemic workplace traps such as unclear authority structures, inconsistent management support, unclear work procedures, and the lack of supporting infrastructure, which make safe handoffs challenging [21]. Such conflicts could be addressed by improving inter-unit teamwork and coordination [25]. Moreover, the provision of expectations and policies from top management that address the assignment of accountability in the delivery of care could reduce delays and improve the coordination of care across unit boundaries. We posit that inter-unit teamwork and a top management that expects and is supportive of patient safety would facilitate effective handoff of unit accountability during patient transitions.

## Methods

### Data

In 2006, the United States Department of Health and Human Services’ (DHHS) Agency for Healthcare Research and Quality (AHRQ) funded the development of the Hospital Survey on Patient Safety Culture (HSOPSC). This survey was administered on a voluntary basis to all hospitals in the United States. The HSOPSC assesses hospital staff opinions on 42 items that measure their institution’s patient safety practices based on 5-point response scales of agreement (“strongly disagree” to “strongly agree”) or frequency (“never” to “always”). The de-identified data for this study comes from the 2010 survey that was made available for public use. It can be requested from the AHRQ. It represents 885 U.S. hospitals that voluntarily participated in the survey [7]. The views of healthcare professionals were aggregated for each institution, since past studies have shown that aggregating these items from the individual- and unit-level responses to the hospital level led to more robust psychometric properties [35], which are reported in Additional file 1.

In Table 1, we report the distribution of respondents by job roles. About two thirds of respondents are from the nursing and allied health professions while another third are administrative staff. A small percentage of respondents were self-identified as physicians, although an unknown percentage of the administrative staff could also be physicians. The responses in this survey are therefore representative of the views of nurses, allied health professionals, management, and physicians.

**Table 1:** Percentage of respondents by job role

Job role	Percentage of respondents
Nurses (RN, PA/NP, LVN/LPN)	37.10 %
Physicians (Attending, Resident)	3.66 %
Allied Healthcare Professionals (Pharmacist, PT, RT, OT, Dietitian, Technicians, Patient Care Assistant)	24.12 %
Staff (Management, Administrative Assistant & other clerical positions)	35.10 %

### Measures

#### Covariates

Four hospital characteristics pertaining to *bedsize, hospital type*, *ownership*, and *staffing* were included as baseline covariates since we expect these factors to systematically affect perceptions of patient safety. For example, large government-owned teaching hospitals may experience more incidents because they serve a more diverse population of patients that present with complex co-morbidities than smaller private specialty hospitals. The frequency distribution for each covariate is reported in Additional file 2.

#### Handoff transfers

Four items related to handoffs and transitions of care in the survey were used for our analyses. *Handoff of patient information* comprises two items, ‘important patient care information is often lost during shift changes’ (reverse coded) and ‘problems often occur in the exchange of information across hospital units’ (reverse coded). *Handoff of personal responsibility in shift changes* is measured by the item, ‘shift changes are problematic for patients in this hospital’ (reverse coded). *Handoff of unit accountability* is measured by the item, ‘things “fall between the cracks” when transferring patients from one unit to another’ (reverse coded).

#### Patient safety culture

Communication culture is measured by two composites, *communication openness* and *feedback and communication about error*. Teamwork culture is measured by two composite scales, *teamwork within units* and *teamwork across units*. Reporting culture is measured by the composite, *frequency of events reported*. Supportive management action is measured by three composites, *management support for patient safety, supervisor/manager expectations and actions promoting patient safety*, and *non-punitive response to error*. The items in the HSOPSC survey that represent each of these composites are reported in Additional file 3.

#### Patient safety perceptions

*Patient safety perceptions* comprises four items that measures respondents’ agreement that ‘patient safety is never sacrificed to get more work done’, ‘our procedures and systems are good at preventing errors from happening’, ‘it is just by chance that more serious mistakes don’t happen around here’ (reverse coded), and ‘we have patient safety problems in this unit’ (reverse coded).

### Statistical analysis

We applied hierarchical multiple linear regression analysis using SPSS v21 to analyze the data. This technique allows us to enter a fixed order of variables to control for the influence of the covariates so that we can isolate the effects of the predictors of patient safety perception. We first entered the four hospital covariates into the regression model as baseline predictors on patient safety perception. We then entered each handoff transfer variable into the regression model. Similarly, to assess the effects of patient safety culture on each handoff transfer, we first entered the four hospital covariates as baseline predictors on each handoff transfer followed by the respective patient safety culture composite.

## Results

First, we check for multicollinearity among the covariates and predictors. Multicollinearity, shown by the variance inflation factor (VIF), results in an inflated variance or R^2^ in the outcome variable in the regression model [36]. In our sample, the VIF was below 3.0, meaning that any significant relationships found are not inflated by correlations between the predictor variables [36]. Table 2 reports strong support for the hypothesis that effective handoffs of information, responsibility, and accountability are statistically significantly (*p* < .001) related to patient safety perceptions.

**Table 2:** Hierarchical regression analyses on the impact of handoffs on patient safety perceptions

	Patient safety perceptions		
	Model 1	Model 2	Model 3
Control variables:			
Bedsize	-.01	.02	.03
Hospital type	-.02	-.04*	-.02
Ownership	-.03	-.05**	-.06**
Staffing	.60***	.62***	.64***
Predictor Variables:			
Handoff of patient information	.35***		
Handoff of personal responsibility		.32***	
Handoff of unit accountability			.32***
Change in R^2^	.069***	.049***	.054***
Total Adj R^2^	.76***	.74***	.745***

Values in the table are standardized beta coefficients for *n* = 885 hospitals

* *p* < .05, ** *p* < .01, *** *p* < .001

Table 3 reports the inter-relationships among handoffs of information, responsibility, and accountability. Model 1 in Table 3 reports that enhancing handoffs of responsibility and unit accountability *enhance* the handoff of patient information. Model 2 in Table 3 explores the relationship between communication culture and the handoff of information. The results in Model 2 shows that while *feedback and communication on error* had a significantly positive effect on perceptions of effective handoff of patient information, *communication openness* had no influence on perceptions of effective handoff of patient information. Thus, a strong communication culture only partially enhances the effective handoff of patient information.

**Table 3:** Hierarchical regression analyses on handoffs

Dependent variables	Handoff of patient information		Handoff of responsibility		Handoff of unit accountability	
	Model 1	Model 2	Model 3	Model 4	Model 5	Model 6
Covariates						
Bedsize	-.13***	-.20***	-.12***	-.01	-.14***	-.02
Hospital Type	-.01	.02	.05**	-.02	-.03	-.02
Ownership	-.06***	.01	.03*	-.01	.05***	-.01
Staffing	.07***	.38***	.15***	.48***	-.01	.46***
Handoff transfer of						
Patient information			.51***		.66***	
Responsibility	.38***				.21***	
Unit accountability	.60***		.25***			
Patient safety culture						
Communication openness		.06				
Feedback & communication on errors		.34***				
Teamwork within units				.15***		
Frequency of events reported				.23***		
Teamwork across units						.74***
Management support for patient safety						.01
Supervisor/Manager expectations & actions promoting patient safety						-.10***
Nonpunitive response to error						.01
Change in R^2^	.420***	.107***	.295***	.078***	.368***	.288***
Total Adj R^2^	.862***	.539***	.813***	.594***	.848***	.768***

Values in the table are standardized beta coefficients for *n* = 885 hospitals

* *p* < .05, ** *p* < .01, *** *p* < .001

Model 3 in Table 3 shows that enhancing handoffs of patient information *and* unit accountability enhance the handoff of responsibility during shift changes. Model 4 in Table 3 shows that both *teamwork within units* and *frequency of events reported* had statistically significant positive influences on perceptions of effective handoff of responsibility in shift changes. Thus, a strong teamwork culture *and* a reporting culture enhance the handoff of responsibility during shift changes.

Model 5 in Table 3 shows that enhancing handoffs of patient information *and* personal responsibility enhance the handoff of unit accountability. Model 6 in Table 3 shows that while *teamwork between units* had a positive and significant association on perceptions of the effective handoff of unit accountability, s*upportive management culture* and *non-punitive response to error* had no effect on the handoff of accountability. We also found that *supervisor/manager expectations and actions promoting patient safety* had a statistically *negative* influence on perceptions of unit accountability. The data indicates that a strong teamwork culture enhances the handoff of unit accountability but this is not in case for management support.

## Discussion

Most handoffs studies have focused on communication issues. They generally recommend structured information handoffs, such as IPASS, as a solution to communication problems. Ours is the first to delineate and empirically test the relationships of three different handoffs in information, responsibility, and accountability on perceptions of patient safety. The results generally show that effective handoffs of patient information, personal responsibility during shift changes, and unit accountability for patient transfers are significantly related to patient safety perceptions. The results also show that each handoff influences the others such that the improvement (or degradation) of one also improves (or erodes) the others. The data shows that communication exchanges, individual behaviors, and organizational processes have to be addressed before shared beliefs and values on perceptions of patient safety can be formed [37].

The results indicate that each type of handoff is affected by different patient safety culture composites. Providing feedback and communication about errors enhanced perceptions of effective handoff of patient information. However, the results indicate that a strong communication culture only *partially* ensures the effective handoff of patient information. Since communication openness is highly correlated with feedback and communication about errors (*r* = 0.63, *p* < 0.01), this finding may be the simple result of measurement since the effect of one cultural composite may mask the effects of the other. Future studies should start with a comprehensive definition of communication culture to include having a minimum data set, the use of mnemonics for communicating relevant information, and a process that include electronic means to support communication.

The data shows that strong teamwork culture and reporting culture enhance *perceptions* of the effective handoff of responsibility during shift changes. Demonstrating such professionalism may require providers to create protected time and space for the handoff during shift change, prepare rationales for plans of care and tasks to perform, and verify that the receiving provider has accurately understood the information received.

The data indicates that providers making the effort to ensure strong teamwork between units by demonstrating cooperation, collaboration, and coordination enhance the handoff of unit accountability. However, it was surprising that management support did not significantly enhance the handoff of unit accountability. Perhaps constant process improvement efforts can create fatigue, so that ‘management support’ is met with cynicism if resources to implement these efforts are insufficient. As well, frontline staff may not observe management support if the former do not routinely interact with the latter. Similarly, *non-punitive responses to error* are not observable if no actions were taken when errors were made. In short, management may need to exhibit the observable appropriate behaviors before unit accountability in handoffs can be enhanced.

The results indicate that we have to focus on specific cultural composites when designing and training healthcare professionals to improve specific types of handoffs. For example, in large hospitals or in complex medical systems, the high workload and the pressures of coordinating clinical care between different units with different experiences and expectations increase challenges to proper handoffs. Here, management may need to invoke the sense of professionalism for all healthcare providers by offering evidence on the causes and consequences of poor handoffs while providing incentives and recognition for performing good handoffs.

The strengths in using the HSOPSC survey data is the large number of hospital participants, which provide robust and stable coefficients in the regression model [38]. The limitations include the following. First, the data is cross-sectional from one time-period. A better estimation technique would be to utilize a panel of data going over several years, but that is not possible because the respondents are anonymous; a different dataset needs to be constructed. Second, physician representation in the data is low and therefore, one cannot generalize the responses or the implications of the results to physicians alone. Steps to incentivize physician participation will need to be taken for the data to represent all stakeholders in the hospital community. Third, no outcomes are reported from this dataset, such as the number of medical errors due to handoffs, the number of close-calls during transitions, or hospital length of stay. Therefore, future studies involving interventions related to handoffs of information, responsibility, and accountability are needed to correlate the implications for handoff practice to actual outcomes as there are none to date. Examples of such interventions may include having a minimum data set when handing over patient information, assessing the efficacy of inter-professional teamwork training on enhancing professionalism, and team-based governance reporting structures to improving unit accountability. Fourth, from a theoretical standpoint, we were limited by the way the constructs were operationalized in the survey and the reliance on self-report data [38]. An opportunity clearly exists to develop comprehensive measures of these constructs in future studies by considering more fine-grained measures of information exchange and communication processes, personal responsibility as it relates to learning and team behaviors as well as unit accountability related to systems improvement, training, and staff empowerment. Having noted all these limitations, we still believe that the study points us toward a richer and theoretically robust way of conceptualizing handoffs.

## Conclusions

The contribution of this study lies in the deconstruction of handoffs into information, responsibility, and accountability and in identifying the accompanying patient safety culture composites that differentially influence each type of handoff. We provided an in-depth look at the cultural drivers of effective handoffs than the literature has thus far examined. The different and sometimes strong cultures between professional specialties can cause the fragmentation of shared values, making it difficult for such professionals to view themselves as part of an organization. If the organization does not have a formal process to help healthcare professionals perceive each other as a resource, the handoff process is carried out in ‘silos’.

In order to help healthcare professionals navigate the tradeoff between efficiency and thoroughness, hospitals can build a strong culture of teamwork across units, while using other organizational development activities to bind its members to a common vision and shared mental model. The theory of planned behavior suggests that attitude is a key factor, which can be influenced by training and education [39]. Perhaps training healthcare professionals with handoffs procedures and protocols can be used to influence a healthcare organization’s patient safety culture. Other techniques include mentoring and leading by example with a sharp focus on transitions of care as a central theme in a hospital’s safety program [40–42]. The interactions between the different types of transitions we showed in this study suggest that spillovers into other aspects of patient safety are likely to occur. More importantly, defining patient safety culture in a specific form (transitions of care) attenuates ambiguity so that stakeholders can more clearly identify with the goals and process of patient safety improvement programs.

## Abbreviations

AHRQ, Agency for Healthcare Research and Quality; DHHS, Department of Health and Human Services (United States); HSOPSC, Hospital Survey on Patient Safety Culture

## Additional files

Additional file 1: Psychometric Properties of the Variables. Descriptive statistics and reliability analyses of the items in each patient safety culture composite. (DOCX 15 kb) Additional file 2: Frequency Distribution of Covariates. The distribution frequency for each covariate (control) variable used in the hierarchical regression model. This is report to describe the sample characteristics. (DOCX 12 kb) Additional file 3: Hospital Survey on Patient Safety Culture (HSOPC) survey items for each Patient Safety Culture Composite. A list of the items and descriptions from the HSOPC used in this study. (DOCX 13 kb)
