# Supplementary material for: Handoffs, safety culture, and practices: evidence from the hospital survey on patient safety culture
Source: BMC Health Serv Res. 2016 Jul 12;16:254. doi: 10.1186/s12913-016-1502-7 (PMC4941024; doi:10.1186/s12913-016-1502-7)
Supplement: Additional file 1: — Psychometric Properties of the Variables. Descriptive statistics and reliability analyses of the items in each patient safety culture composite. (DOCX 15 kb) [file 12913_2016_1502_MOESM1_ESM.docx]

**Additional file 1: Psychometric Properties of the Variables**

We perform reliability analyses on the items that represent each patient safety culture composite to ensure that the items are theoretically similar in each composite. The Cronbach's alpha for each composite as shown on Table 1 range from 0.71 to 0.87, showing acceptable reliability of at least .70 [43].

**Descriptive Statistics^1^**

|  | Mean | s.d. | 1 | 2 | 3 | 4 | 5 | 6 | 7 | 8 | 9 | 10 | 11 | 12 |
| --- | --- | --- | --- | --- | --- | --- | --- | --- | --- | --- | --- | --- | --- | --- |
| 1. Patient Safety Perceptions | 3.59 | .78 | (.72)^2^ |  |  |  |  |  |  |  |  |  |  |  |
| 2. Handoff of Unit Accountability | 3.03 | 1.01 | .37 |  |  |  |  |  |  |  |  |  |  |  |
| 3. Handoff of Personal Responsibility | 3.19 | 1.00 | .36 | .47 |  |  |  |  |  |  |  |  |  |  |
| 4. Handoff of Patient Information | 3.22 | .87 | .41 | .64 | .59 | (.74) |  |  |  |  |  |  |  |  |
| 5. Management Support for Patient Safety | 3.74 | .82 | .59 | .39 | .37 | .42 | (.78) |  |  |  |  |  |  |  |
| 6. Supervisor/Manager Expectations & Actions Promoting Patient Safety | 3.86 | .79 | .53 | .26 | .26 | .31 | .52 | (.78) |  |  |  |  |  |  |
| 7. Non-punitive Response to Error | 3.15 | .91 | .46 | .26 | .27 | .31 | .37 | .46 | (.79) |  |  |  |  |  |
| 8. Communication Openness | 3.66 | .81 | .49 | .28 | .27 | .32 | .46 | .57 | .49 | (.71) |  |  |  |  |
| 9. Feedback & Communication about Errors | 3.74 | .84 | .48 | .28 | .26 | .31 | .50 | .56 | .36 | .63 | (.79) |  |  |  |
| 10. Frequency of Events Reported | 3.71 | .96 | .36 | .24 | .22 | .26 | .36 | .33 | .23 | .39 | .47 | (.87) |  |  |
| 11. Teamwork Within Units | 3.92 | .78 | .48 | .24 | .25 | .29 | .41 | .48 | .39 | .50 | .46 | .29 | (.84) |  |
| 12. Teamwork Across Units | 3.42 | .76 | .45 | .57 | .47 | .59 | .56 | .39 | .34 | .41 | .41 | .30 | .42 | (.79) |

^1^All bivariate correlations are significant at p < 0.01 for n = 885 hospitals

^2^Values in parenthesis are Cronbach’s alphas
