# Supplementary material for: Handoffs, safety culture, and practices: evidence from the hospital survey on patient safety culture
Source: BMC Health Serv Res. 2016 Jul 12;16:254. doi: 10.1186/s12913-016-1502-7 (PMC4941024; doi:10.1186/s12913-016-1502-7)
Supplement: Additional file 2: — Frequency Distribution of Covariates. The distribution frequency for each covariate (control) variable used in the hierarchical regression model. This is report to describe the sample characteristics. (DOCX 12 kb) [file 12913_2016_1502_MOESM2_ESM.docx]

**Additional file 2: Frequency Distribution of Covariates**

| Bedsize: | Frequency (number of hospitals) |
| --- | --- |
| 1 = 6-24 beds | 73 |
| 2 = 25-49 beds | 161 |
| 3 = 50-99 beds | 151 |
| 4 = 100-199 beds | 188 |
| 5 = 200-299 beds | 129 |
| 6 = 300-399 beds | 70 |
| 7 = 400-499 beds | 47 |
| 8 = 500 or more beds | 65 |
| Hospital Type: |  |
| 0 = Non-teaching | 599 |
| 1 = Teaching | 285 |
| Ownership: |  |
| 0 = Non-government | 712 |
| 1 = Government | 172 |
| Staffing Composite | Mean = 3.44, s.d. = .25, alpha = .61 |
