# Supplementary material for: Handoffs, safety culture, and practices: evidence from the hospital survey on patient safety culture
Source: BMC Health Serv Res. 2016 Jul 12;16:254. doi: 10.1186/s12913-016-1502-7 (PMC4941024; doi:10.1186/s12913-016-1502-7)
Supplement: Additional file 3: — Hospital Survey on Patient Safety Culture (HSOPC) survey items for each Patient Safety Culture Composite. A list of the items and descriptions from the HSOPC used in this study. (DOCX 13 kb) [file 12913_2016_1502_MOESM3_ESM.docx]

**Additional file 3: Hospital Survey on Patient Safety Culture survey items for each Patient Safety Culture Composite**

Communication Openness

1. Staff will freely speak up if they see something that may negatively affect patient care.

2. Staff feel free to question the decisions or actions of those with more authority.

3. Staff are afraid to ask questions when something does not seem right. (reverse coded)

Feedback & Communication About Error

1. We are given feedback about changes put into place based on event reports.

2. We are informed about errors that happen in this unit.

3. In this unit, we discuss ways to prevent errors from happening again.

Teamwork Within Units

1. People support one another in this unit.

2. When a lot of work needs to be done quickly, we work together as a team to get the work done.

3. In this unit, people treat each other with respect.

4. When one area in this unit gets really busy, others help out.

Frequency of Events Reported

1. When a mistake is made, but is caught and corrected before affecting the patient, how often is this reported?

2. When a mistake is made, but has no potential to harm the patient, how often is this reported?

3. When a mistake is made that could harm the patient, but does not, how often is this reported?

Teamwork Across Units

1. There is good cooperation among hospital units that need to work together.

2. Hospital units work well together to provide the best care for patients.

3. Hospital units do not coordinate well with each other. (reverse coded)

4. It is often unpleasant to work with staff from other hospital units. (reverse coded)

Management Support for Patient Safety

1. Hospital management provides a work climate that promotes patient safety.

2. The actions of hospital management show that patient safety is a top priority.

3. Hospital management seems interested in patient safety only after an adverse event happens. (reverse coded)

Supervisor/Manager Expectations & Actions Promoting Patient Safety

1. My supervisor/manager says a good word when he/she sees a job done according to established patient safety procedures.

2. My supervisor/manager seriously considers staff suggestions for improving patient safety.

3. Whenever pressure builds up, my supervisor/manager wants us to work faster, even if it means taking shortcuts. (reverse coded)

4. My supervisor/manager overlooks patient safety problems that happen over and over. (reverse coded)

Non-punitive Response to Errors

1. Staff feel like their mistakes are held against them. (reverse coded)

2. When an event is reported, it feels like the person is being written up, not the problem.

(reverse coded)

3. Staff worry that mistakes they make are kept in their personnel file. (reverse coded)

Staffing

1. We have enough staff to handle the workload.

2. Staff in this unit work longer hours than is best for patient care. (reverse coded)

3. We use more agency/temporary staff than is best for patient care. (reverse coded)

4. We work in "crisis mode" trying to do too much, too quickly. (reverse coded)
